# P034: Daily observation rounds to assess the practice for patients isolated because of diarrhea

**DOI:** 10.1186/2047-2994-2-S1-P34

**Published:** 2013-06-20

**Authors:** R Mikkelsen, B Kure, K Schønning

**Affiliations:** 1 Clinical Microbiologi, Hvidovre Hospital, Hvidovre, Denmark; 2 Internal Medicin, Bornholms Hospital, Rønne, Denmark

## Introduction

The study was conducted at Bornholms Hospital located on an island in the Baltic Sea. In 2011, cases with *Clostridium difficile* infection (CDI) doubled to 50 cases. Cases of ribotype 027 increased 5-fold from 5 to 26. In 2012, multiple interventions were initiated reduce the number of CDI cases.

## Methods

Observation rounds during weekdays seeing patients isolated because of diarrhea was introduced February 1, 2012 in 3 medical wards (94 beds). The rounds adjusted incorrect interventions and collected data structured by checkpoints that included:

· Indication for and duration of isolation

· Turnaround time for microbiological diagnosis

· Antibiotic (AB) therapy prior to episode of diarrhea

· Compliance to isolation precautions

Data were censored January 31, 2013.

## Results

CDI cases decreased to 25 compared to 50 the preceding year (ribotype 027; 3 from 26).In the study period, 100 patients were isolated resulting in a total of 486 isolation days. In all, 20 tested positive, of these 3 with ribotype 027. CDI accounted for a total of 173 isolation days. Average duration of isolation for patients with negative microbiologicial test results was 4.6 days.

Average turnaroundtime was 3.3 days; transportation to laboratory accounted for 2.2 days.

55/100 patients had received AB therapy within 2 months prior to the episode; these included all patients with microbiologically verifiedCDI (RR 3.88; P<0.001).

Compliance with isolation precautions was high throughout the study: proper signage 95%, proper use of protective gear 95%, and proper waste management 95%.

## Conclusion

Daily rounds to audit implementation of isolation precautions documented strict adherence to guidelines during the study period. This may have contributed to the observed decrease in number of CDI.

Only 35% of isolation days were implemented for patients with CDI. Information on prior AB treatment could be a useful decision parameter to implement isolation precuations.

Point of Care testing to eliminate transportation time to laboratory across sea could reduce turnaround time.

Both actions may reduce the number of isolation days unrelated to CDI.

## Disclosure of interest

None declared

